# Supplementary material for: Topological isolation of developmental regulators in mammalian genomes
Source: Nat Commun. 2021 Aug 12;12:4897. doi: 10.1038/s41467-021-24951-7 (PMC8361032; doi:10.1038/s41467-021-24951-7)
Supplement: Supplementary file 2 — Description of Additional Supplementary Files [file 41467_2021_24951_MOESM2_ESM.pdf]

Description of Additional Supplementary Files

File name: Supplementary Data 1.
Description: Data summary of HUES64 Hi-C.

File name: Supplementary Data 2.
Description: CTCF loop domains in HuES64.

File name: Supplementary Data 3.
Description: Capture Hi-C summary.

File name: Supplementary Data 4.
Description: Constitutive CTCF loop domain.

File name: Supplementary Data 5.
Description: Datasets used in this study.
