# Supplementary material for: Topological isolation of developmental regulators in mammalian genomes
Source: Nat Commun. 2021 Aug 12;12:4897. doi: 10.1038/s41467-021-24951-7 (PMC8361032; doi:10.1038/s41467-021-24951-7)
Supplement: Supplementary file 8 — Supplementary Information [file 41467_2021_24951_MOESM8_ESM.pdf]

Topological isolation of developmental regulators in mammalian genomes

Hua-Jun Wu, Alexandro Landshammer, Elena Stamenova, Adriano Bolondi, Helene Kretzmer, Alexander Meissner, and Franziska Michor

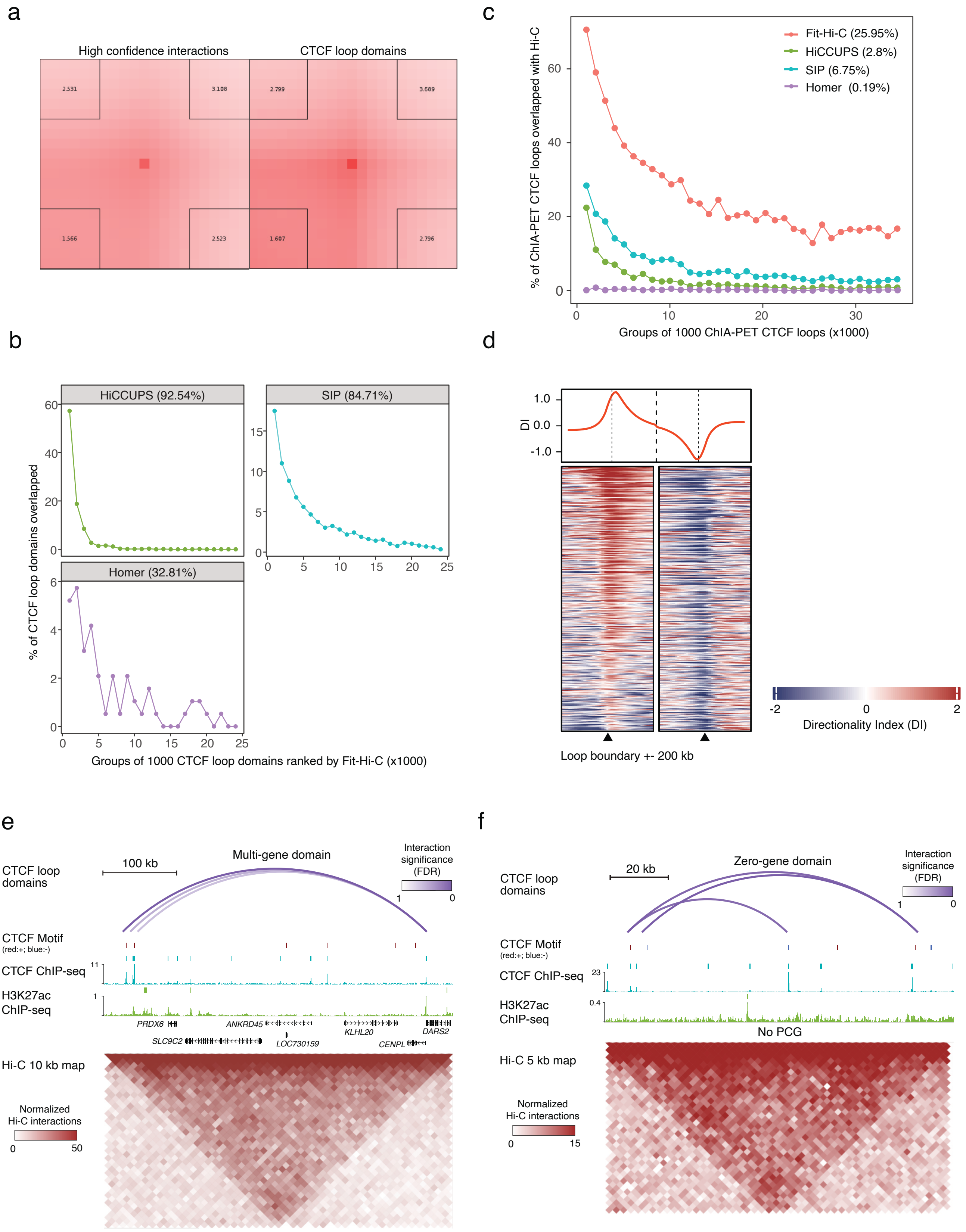
a
High confidence interactions
CTCF loop domains
2.531
3.108
1.566
2.523
2.799
3.689
1.607
2.796
b
HiCCUPS (92.54%)
SIP (84.71%)
Homer (32.81%)
% of CTCF loop domains overlapped
Groups of 1000 CTCF loop domains ranked by Fit-Hi-C (x1000)
c
Fit-Hi-C (25.95%)
HiCCUPS (2.8%)
SIP (6.75%)
Homer (0.19%)
% of ChIA-PET CTCF loops overlapped with Hi-C
Groups of 1000 ChIA-PET CTCF loops (x1000)
d
DI
Loop boundary +- 200 kb
Directionality Index (DI)
e
CTCF loop domains
100 kb
Multi-gene domain
Interaction significance (FDR)
CTCF Motif
(red:+; blue:-)
CTCF ChIP-seq
H3K27ac ChIP-seq
PRDX6
ANKRD45
KLHL20
DARS2
SLC9C2
LOC730159
CENPL
Hi-C 10 kb map
Normalized Hi-C interactions
f
CTCF loop domains
20 kb
Zero-gene domain
Interaction significance (FDR)
CTCF Motif
(red:+; blue:-)
CTCF ChIP-seq
H3K27ac ChIP-seq
No PCG
Hi-C 5 kb map
Normalized Hi-C interactions

Supplementary Fig. 1: Identification and classification of CTCF loop domains in HUES64 cell line.

(a) Aggregate peak analysis (APA) plot for 231,970 high confidence interactions and 24,056 CTCF loop domains called from HUES64 Hi-C data. The APA score (number in the four corners outlined by the black box) is the ratio of the number of contacts in the central bin to the mean number of contacts in the corner.

(b) Comparisons of CTCF loop domains identified by Fit-Hi-C and other methods. The percent of CTCF loop domains overlapping between Fit-Hi-C and HiCCUPS, SIP, Homer in HUES64 Hi-C data are plotted for loops ranked from the most significant to the least significant by Fit-Hi-C. Each point represents 1000 loops. The total percentage of CTCF loop domains overlapping for each method is shown in parentheses on top of each subplot. The CTCF loop domains are defined to be exactly the same without any shift in this analysis.

(c) The percent of insulated neighborhoods identified by ChIA-PET overlapping with CTCF loop domains obtained by different methods in Hi-C data are plotted for insulated neighborhoods ranked by ChIA-PET data from the most significant to the least significant. The total percentages of insulated neighborhoods overlapping with different Hi-C methods are shown in parentheses in the legend. Each point represents 1000 loops. Insulated neighborhoods in ChIA-PET were obtained from the supplementary table of the original paper.

(d) Directionality Indices (DI) obtained from 10 kb Hi-C map in HUES64 cells by Homer are plotted for the surrounding regions (200 kb upstream and downstream) of the left and right CTCF loop domain boundaries to depict their insulation function. The left heatmap shows the surrounding regions of the left boundary, and the right heatmap shows the surrounding regions of the right boundary. Each row represents one CTCF loop domain. Black arrow heads represent the left and right boundaries. The average profiles are shown on top of the heatmap. Higher signal in the left heatmap represents entering an insulated region, and lower signal in the right heatmap represents exiting an insulated region.

(e) Display of a multi-gene CTCF loop domain at *PRDX6* locus. Tracks are shown in the same way as in **Fig. 1c**, except each pixel in Hi-C plot represents 5-kb genomic region.

(f) Display of zero-gene CTCF loop domain at chr1:5680000-5920000. Tracks are shown in the same way as in **Fig. 1c**, except each pixel in Hi-C plot represents 5-kb genomic region.

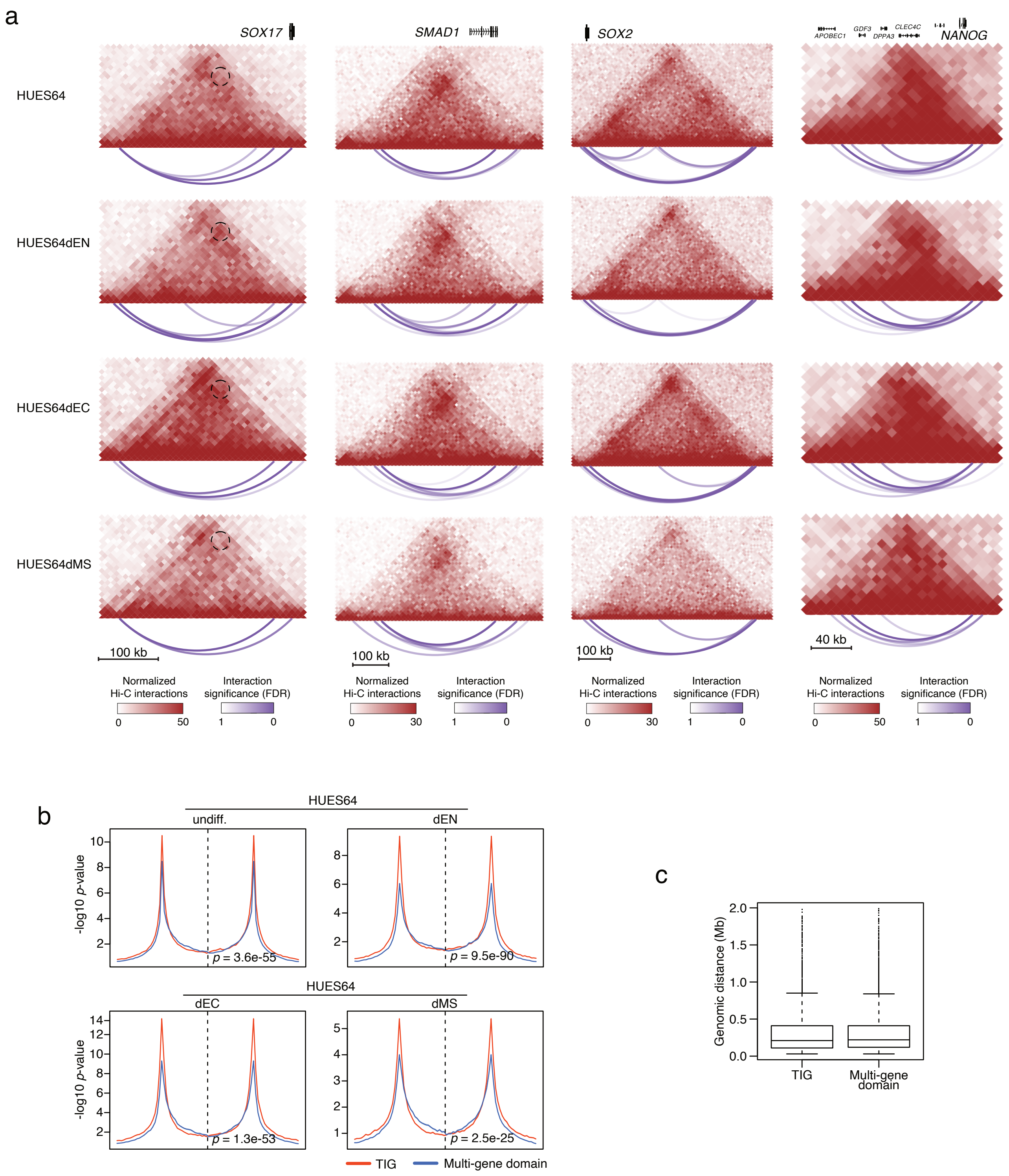
a
SOX17
SMAD1
SOX2
APOBEC1
GDF3
DPPA3
CLEC4C
NANOG
HUES64
HUES64dEN
HUES64dEC
HUES64dMS
100 kb
40 kb
Normalized Hi-C interactions
Interaction significance (FDR)
0
50
30
1
b
HUES64
undiff.
dEN
dEC
dMS
-log10 p-value
p = 3.6e-55
p = 9.5e-90
p = 1.3e-53
p = 2.5e-25
TIG
Multi-gene domain
c
Genomic distance (Mb)
2.0
1.5
1.0
0.5
0.0
TIG
Multi-gene domain

Supplementary Fig. 2: Boundaries of CTCF loop domains.

(a) Preserved pattern of CTCF loop domains during ES differentiation exemplified at *SOX17*, *SMAD1*, *SOX2* and *NANOG* loci. Normalized Hi-C interactions are shown as a heatmap with each pixel representing a 10-kb genomic region. The interaction significance (FDR) was calculated from Hi-C data, and is shown as arcs. Circles in the *SOX17* locus represent the interactions between *SOX17* promoter and distal enhancers induced in endoderm.

(b) Boundary-anchored virtual 4-C average profile of the CTCF loop domain boundaries in HUES64 and its derivatives. The locations of CTCF loop domain boundaries were identified in HUES64 Hi-C data. The -log10 p-values obtained from Fit-Hi-C data are shown. The dotted line separates the left and right boundary regions, which represent the regions in the left and right heatmap in Fig. 2A. Average signals across all boundaries are shown with the shaded area indicating the standard error. Two-sided Wilcoxon test was used to determine the significance level of boundary-to-boundary interactions between the two groups. Data are presented as mean values +/- SE.

(c) The distribution of genomic distances spanning CTCF loop domains of TIGs and nonTIGs. The box indicates the interquartile range (IQR), the line inside the box shows the median, and whiskers show the locations of either 1.5 × IQR above the third quartile or 1.5 × IQR below the first quartile, n = 3310 boundary-to-boundary interactions for TIG, n = 8729 boundary-to-boundary interactions for multi-gene domain.

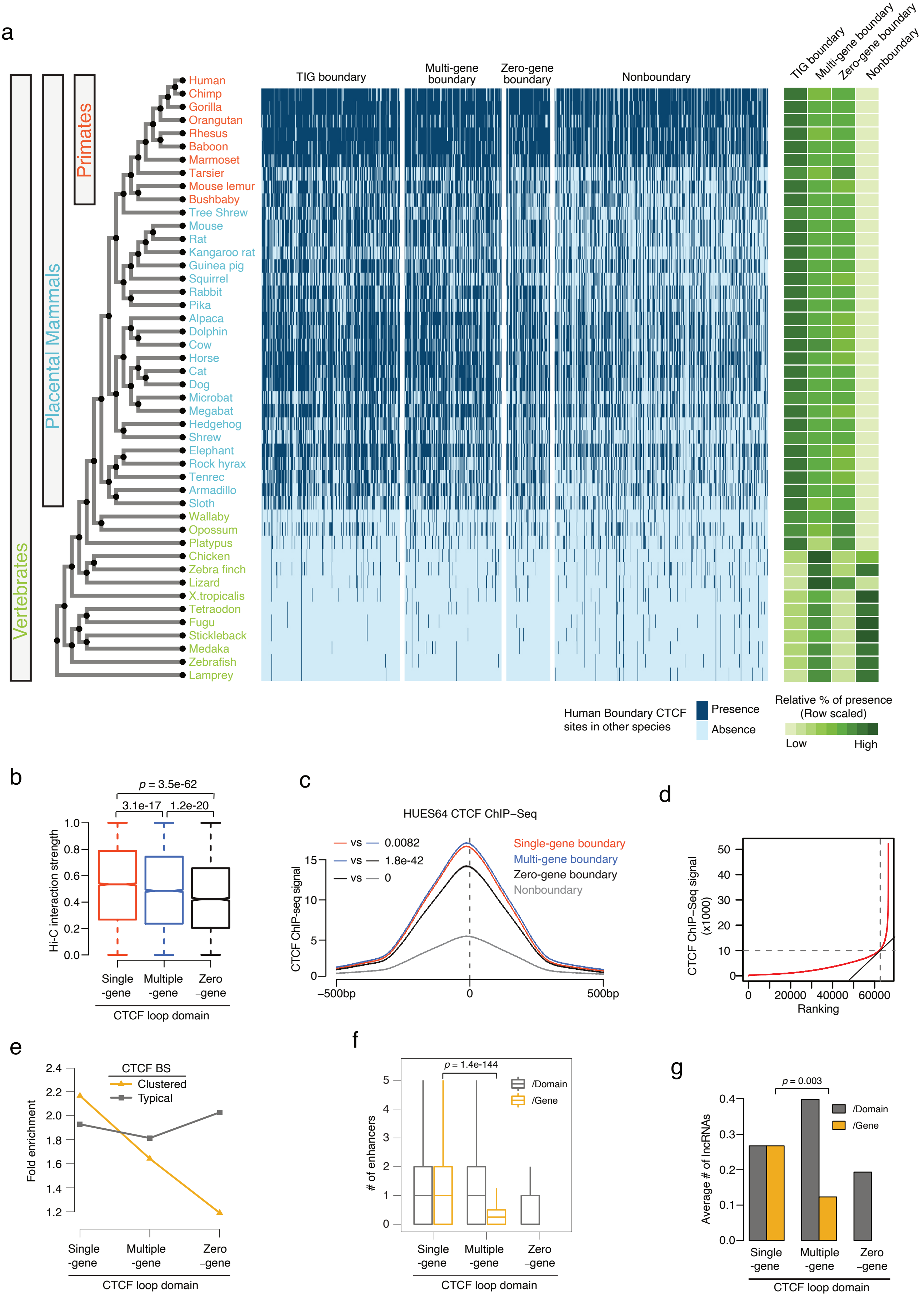
a
Vertebrates
Placental Mammals
Primates
Human
Chimp
Gorilla
Orangutan
Rhesus
Baboon
Marmoset
Tarsier
Mouse lemur
Bushbaby
Tree Shrew
Mouse
Rat
Kangaroo rat
Guinea pig
Squirrel
Rabbit
Pika
Alpaca
Dolphin
Cow
Horse
Cat
Dog
Microbat
Megabat
Hedgehog
Shrew
Elephant
Rock hyrax
Tenrec
Armadillo
Sloth
Wallaby
Opossum
Platypus
Chicken
Zebra finch
Lizard
X.tropicalis
Tetraodon
Fugu
Stickleback
Medaka
Zebrafish
Lamprey
TIG boundary
Multi-gene boundary
Zero-gene boundary
Nonboundary
TIG boundary
Multi-gene boundary
Zero-gene boundary
Nonboundary
Human Boundary CTCF sites in other species
Presence
Absence
Relative % of presence (Row scaled)
Low
High
b
p = 3.5e-62
3.1e-17
1.2e-20
Hi-C interaction strength
1.0
0.8
0.6
0.4
0.2
0.0
Single -gene
Multiple -gene
Zero -gene
CTCF loop domain
c
HUES64 CTCF ChIP–Seq
vs 0.0082
vs 1.8e-42
vs 0
Single-gene boundary
Multi-gene boundary
Zero-gene boundary
Nonboundary
CTCF ChIP-seq signal
15
10
5
0
−500bp
0
500bp
d
CTCF ChIP–Seq signal (x1000)
50
40
30
20
10
0
0
20000
40000
60000
Ranking
e
CTCF BS
Clustered
Typical
Fold enrichment
2.4
2.2
2.0
1.8
1.6
1.4
1.2
Single -gene
Multiple -gene
Zero −gene
CTCF loop domain
f
p = 1.4e-144
/Domain
/Gene
# of enhancers
5
4
3
2
1
0
Single -gene
Multiple -gene
Zero −gene
CTCF loop domain
g
p = 0.003
/Domain
/Gene
Average # of lncRNAs
0.4
0.3
0.2
0.1
0.0
Single -gene
Multiple -gene
Zero −gene
CTCF loop domain

Supplementary Fig. 3: Characterization of different types of CTCF loop domains.

(a) Presence or absence of human CTCF motifs in 45 vertebrates. CTCF motifs were grouped based on the CTCF loop domain classification in HUES64 cell line. The relative proportion of present motifs in each group per species were calculated and shown in the right panel. Specifically, the absolute proportion of present motifs was calculated and row *z*-scored to obtain the relative proportion of present motifs. The phylogenetic tree is obtained from UCSC genome browser.

(b) The strength of Hi-C interactions in different types of domains. The significance (-log10 p value) of Hi-C interaction is rank normalized and displayed in y-axis with "1" represents most significant interactions and "0" represents least significant interactions. The box indicates the interquartile range (IQR), the line inside the box shows the median, and whiskers show the locations of either 1.5 × IQR above the third quartile or 1.5 × IQR below the first quartile, n = 9673 single-gene domains, n = 9189 multiple-gene domains, n = 5194 zero-gene domains. P values calculated by two-sided Wilcoxon test.

(c) CTCF ChIP-Seq signal of consensus CTCF motifs at boundaries of single-gene, multi-gene, and zero-gene CTCF loop domains and nonboundaries. See other descriptions in Fig. 3C.

(d) CTCF ChIP-seq signal of stitched peaks (y-axis) is plotted against the rank of the signal from low to high. Transition point was identified as described in Methods. Peaks above the point is clustered CTCF sites, while peaks below the point is typical CTCF sites.

(e) Fold enrichment of clustered and typical CTCF binding sites (CTCF BS) in the boundaries of different types of domains. Clustered CTCF binding sites have broader and more CTCF binding based on the ChIP-seq experiment.

(f) The number of enhancers per domain (or per gene) within different types of domains. Grey represents the number of features per domain, and orange represents the number of features per gene. The box indicates the interquartile range (IQR), the line inside the box shows the median, and whiskers show the locations of either 1.5 × IQR above the third quartile or 1.5 × IQR below the first quartile, n = 9673 single-gene domains, n = 9189 multiple-gene domains, n = 5194 zero-gene domains. P values calculated by two-sided Wilcoxon test.

(g) The average number of long non-coding RNAs (lncRNAs) within different types of domains. P values calculated by two-sided Wilcoxon test.

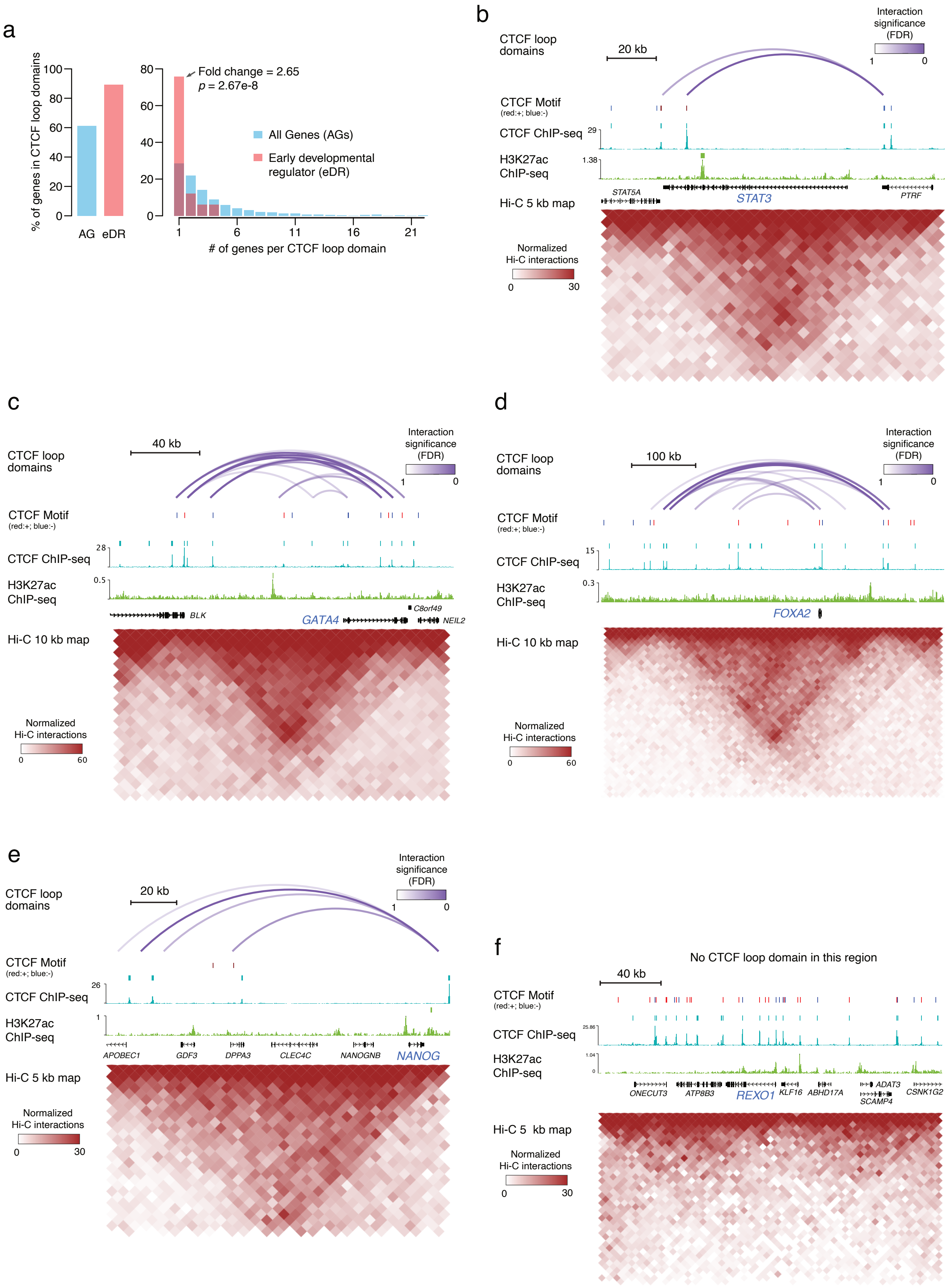
a
% of genes in CTCF loop domains
AG
eDR
Fold change = 2.65
p = 2.67e-8
All Genes (AGs)
Early developmental regulator (eDR)
# of genes per CTCF loop domain
b
CTCF loop domains
20 kb
Interaction significance (FDR)
CTCF Motif (red:+; blue:-)
CTCF ChIP-seq
H3K27ac ChIP-seq
Hi-C 5 kb map
STAT5A
STAT3
PTRF
Normalized Hi-C interactions
c
40 kb
BLK
GATA4
C8orf49
NEIL2
Hi-C 10 kb map
d
100 kb
FOXA2
e
APOBEC1
GDF3
DPPA3
CLEC4C
NANOGNB
NANOG
f
No CTCF loop domain in this region
ONECUT3
ATP8B3
REXO1
KLF16
ABHD17A
ADAT3
SCAMP4
CSNK1G2

Supplementary Fig. 4: Early developmental regulators in CTCF loop domains.

(a) Enrichment of early developmental regulators in single-gene domains. The left panel represents the percent of all genes (AG) or early developmental regulators (eDR) located within domains. The right panel represents the percent of AG or eDR located within domains with increasing number of protein coding genes. P-values calculated by two-sided Fisher's exact test.

(b) Display of genomic locus at *STAT3* as a TIG that is in a single-gene domain. Domains displayed as arcs overlayed on top of CTCF and H3K27ac ChIP-seq profiles and normalized Hi-C interaction map. CTCF peaks and enhancers are denoted as bars above the ChIP-seq profiles of CTCF and H3K27ac. CTCF consensus motifs are denoted as red (forward orientation) and blue (reverse orientation) bars above the CTCF peaks. Normalized Hi-C interactions are denoted as heatmap with each pixel represents 5-kb genomic region. The interaction significance (FDR) was calculated from Hi-C data.

(c) Display of genomic locus at *GATA4* as a TIG that is in a single-gene domain. Tracks are shown in the same way as in (**a**).

(d) Display of genomic locus at *FOXA2* as a TIG that is in a single-gene domain. Tracks are shown in the same way as in (**a**).

(e) Display of genomic locus at *NANOG* that is in a multi-gene domain. Tracks are shown in the same way as in (**a**).

(f) Display of genomic locus at *REXO1* that is not in a domain. Tracks are shown in the same way as in (**a**).

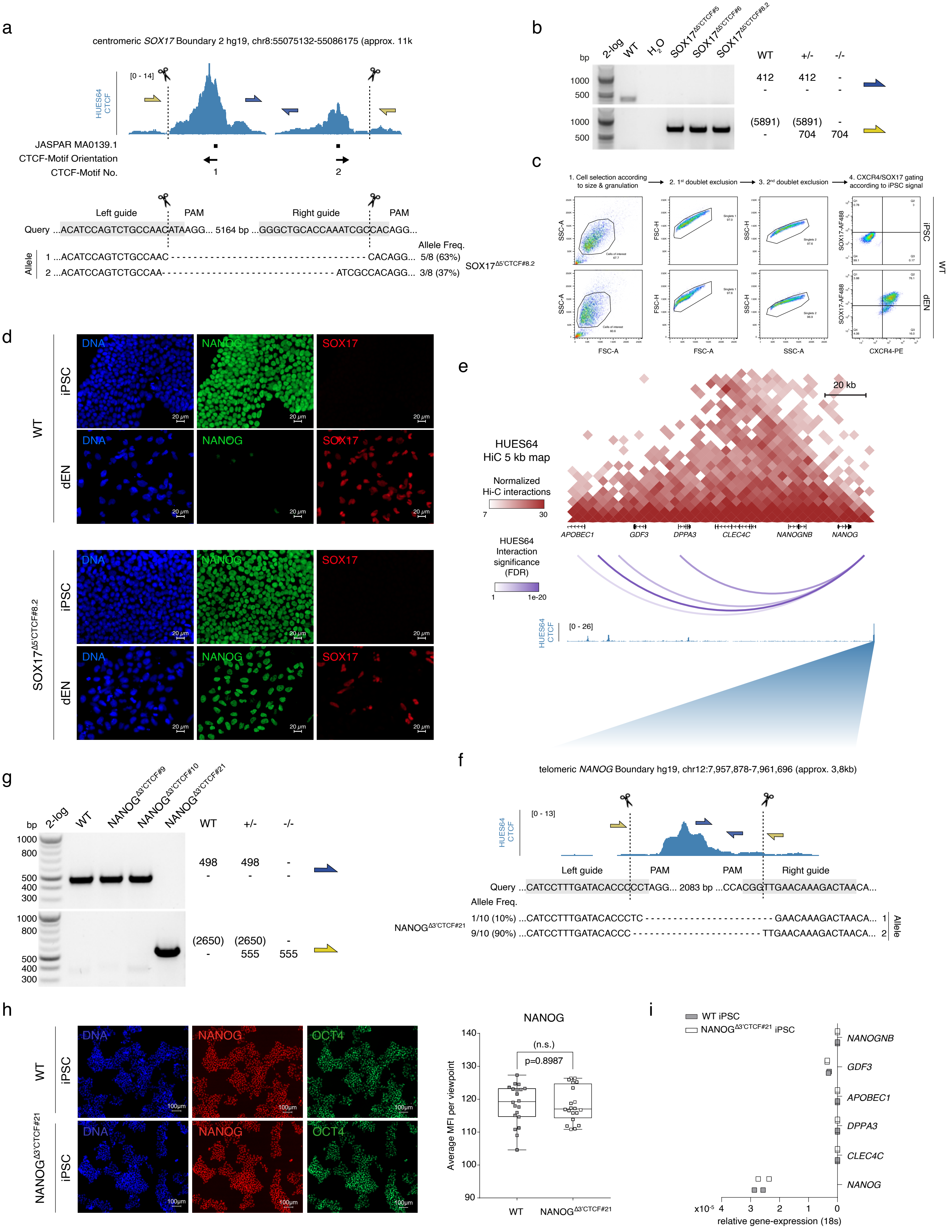
a
centromeric SOX17 Boundary 2 hg19, chr8:55075132-55086175 (approx. 11k
HUES64 CTCF
[0 - 14]
JASPAR MA0139.1
CTCF-Motif Orientation
CTCF-Motif No.
1
2
Left guide
PAM
Right guide
PAM
Query ...ACATCCAGTCTGCCAACATAAGG... 5164 bp ...GGGCTGCACCAAATCGCCACAGG...
Allele Freq.
Allele
1 ...ACATCCAGTCTGCCAAC --------- CACAGG... 5/8 (63%)
2 ...ACATCCAGTCTGCCAA- --------- ATCGCCACAGG... 3/8 (37%)
SOX17Δ5'CTCF#8.2
b
bp
2-log
WT
H2O
SOX17Δ5'CTCF#5
SOX17Δ5'CTCF#6
SOX17Δ5'CTCF#8.2
1000
500
WT
+/-
-/-
412
412
-
(5891)
(5891)
704
704
c
1. Cell selection according to size & granulation
2. 1st doublet exclusion
3. 2nd doublet exclusion
4. CXCR4/SOX17 gating according to iPSC signal
SSC-A
FSC-H
SSC-H
SOX17-AF488
FSC-A
SSC-A
CXCR4-PE
iPSC
dEN
WT
d
WT
iPSC
dEN
SOX17Δ5'CTCF#8.2
DNA
NANOG
SOX17
20 μm
e
HUES64
HiC 5 kb map
Normalized
Hi-C interactions
7
30
20 kb
APOBEC1
GDF3
DPPA3
CLEC4C
NANOGNB
NANOG
HUES64
Interaction
significance
(FDR)
1
1e-20
HUES64 CTCF
[0 - 26]
f
telomeric NANOG Boundary hg19, chr12:7,957,878-7,961,696 (approx. 3,8kb)
HUES64 CTCF
[0 - 13]
Left guide
PAM
PAM
Right guide
Query ...CATCCTTTGATACACCCCTAGG... 2083 bp ...CCACGGTTGAACAAAGACTAACA...
Allele Freq.
NANOGΔ3'CTCF#21
1/10 (10%) ...CATCCTTTGATACACCCTC --------- GAACAAAGACTAACA... 1
9/10 (90%) ...CATCCTTTGATACACCC --------- TTGAACAAAGACTAACA... 2
Allele
g
bp
2-log
WT
NANOGΔ3'CTCF#9
NANOGΔ3'CTCF#10
NANOGΔ3'CTCF#21
1000
800
500
400
300
WT
+/-
-/-
498
498
(2650)
(2650)
555
555
h
WT
iPSC
NANOGΔ3'CTCF#21
DNA
NANOG
OCT4
100μm
NANOG
(n.s.)
p=0.8987
Average MFI per viewpoint
140
130
120
110
100
90
WT
NANOGΔ3'CTCF#21
i
WT iPSC
NANOGΔ3'CTCF#21 iPSC
NANOGNB
GDF3
APOBEC1
DPPA3
CLEC4C
NANOG
x10-5 4 3 2 1 0
relative gene-expression (18s)

Supplementary Fig. 5: Phenotype of *SOX17* TIG boundary, and *NANOG* CTCF loop domain boundary perturbation.

(a) Centromeric *SOX17* boundary ($SOX17^{\Delta 5'CTCF}$) targeting strategy scheme. Scissors and dashed lines indicate CRISPR/Cas9 target-sites. Representative chromatogram-data including allele-frequency of sanger-sequenced, pJET1.2 cloned PCR-products of clone $SOX17^{\Delta 5'CTCF\#8.2}$ are depicted below.

(b) Expected PCR-band pattern for different primer-pairs of centromeric *SOX17* boundary targeting are indicated by primer color. 1% agarose-gel pictures depict genotyping PCR band size-separations of respective knockout clone and control gDNA.

(c) FlowJo gating strategy example. Cells were selected according to size and granulation (1) followed by single cell selection (2-3). In case of SOX17/CXCR4, cell population percentages considered positive (4) are based on cell selections according to iPSC signal.

(d) Immunofluorescent stainings of NANOG, SOX17 and DNA (DAPI) from day 0/5 *in vitro* endoderm differentiated $SOX17^{\Delta 5'CTCF\#8.2}$ or wild type cells.

(e) Multi-layered display of the *NANOG* locus as a representative CTCF loop domain (multi-gene domain) at chr12: 7810000-7960000. HUES64 CTCF loop domains are displayed as arcs below a normalized Hi-C interaction map following by CTCF ChIP-seq profiles.

(f) Telomeric *NANOG* boundary ($NANOG^{\Delta 3'CTCF}$) targeting strategy scheme. Targeting regions for the sgRNAs are depicted at the CTCF ChIP-seq profile indicated by dashed lines and scissors for the predicted Cas9 cut sites. Representative chromatogram-data including allele-frequency of sanger-sequenced, pJET1.2 cloned PCR-products of clone $NANOG^{\Delta 3'CTCF\#21}$ are depicted below.

(g) Expected PCR-band pattern for different primer-pairs of telomeric *NANOG* boundary targeting are indicated by primer color. 1% agarose-gel pictures depict genotyping PCR band size-separations of respective knockout clone and control gDNA.

(h) Immunofluorescent stainings of NANOG, OCT4 (POU5F1) and DNA (DAPI) from undifferentiated iPSC of $NANOG^{\Delta 3'CTCF\#21}$ or wild type cells. Additionally, NANOG Mean fluorescence intensity (MFI) calculated in $NANOG^{\Delta 3'CTCF\#21}$ and wild type iPSCs (n=10 independent regions (regional MFI average per cell) over 2 experiments with WT=20312 and $NANOG^{\Delta 3'CTCF\#21}$=17119 total cells). Statistical significance was determined by a two-tailed Mann Whitney U test. Data are presented as box plots, where the median is depicted as the box center, and 25% and 75% quartile are indicated as box bounds between whiskers that highlight minimum and maximum.

(i) qRT-PCR of bulk-populations from genes associated within the *NANOG* CTCF loop domain. Expression values are depicted as relative gene-expression ($2^{-(\Delta Ct(GOI-18s))}$) (n=2 biological replicates).

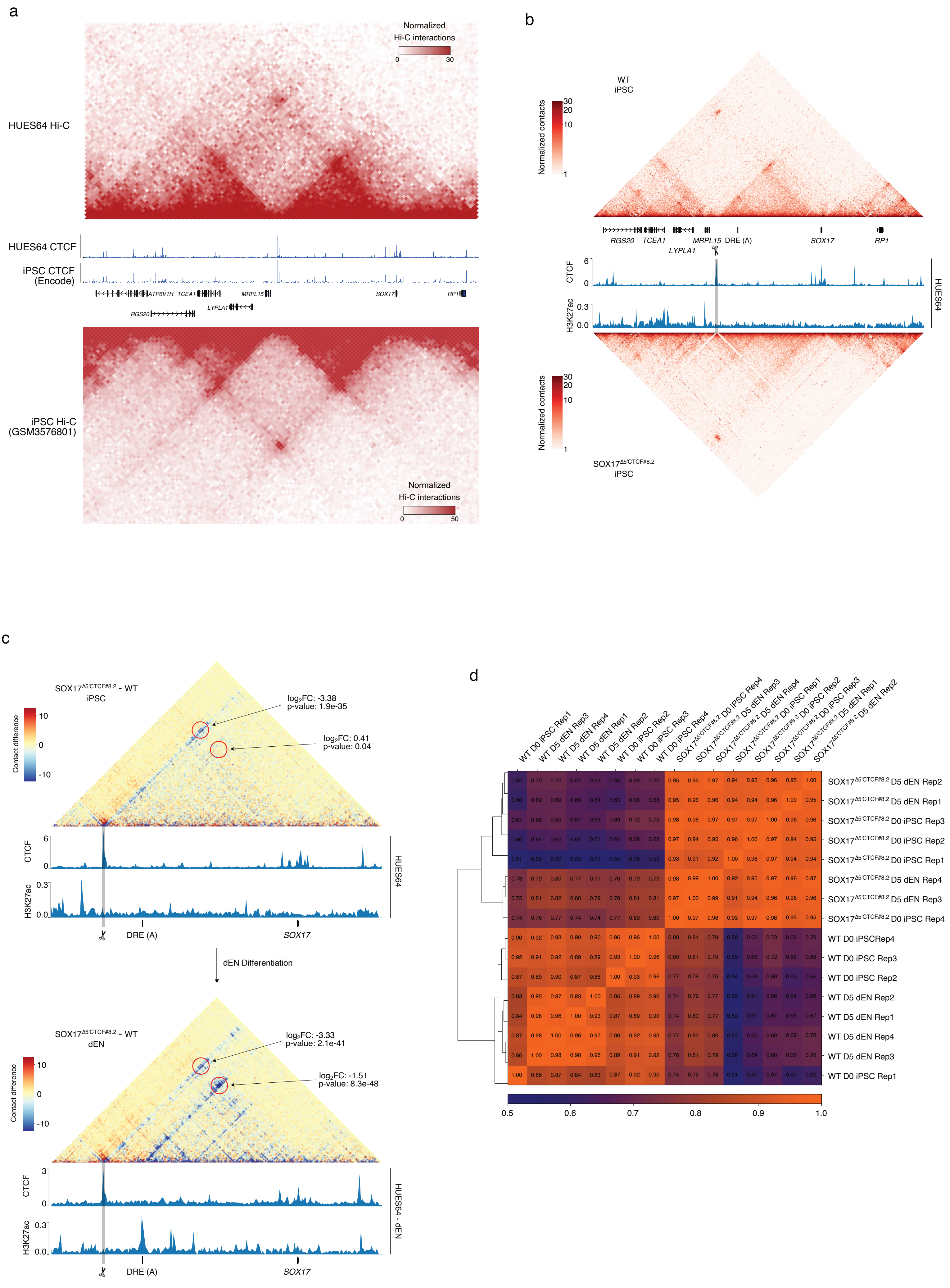
a
HUES64 Hi-C
Normalized
Hi-C interactions
0
30
HUES64 CTCF
iPSC CTCF
(Encode)
ATP6V1H
TCEA1
MRPL15
SOX17
RP1
RGS20
LYPLA1
iPSC Hi-C
(GSM3576801)
Normalized
Hi-C interactions
0
50
b
WT
iPSC
Normalized contacts
30
20
10
1
RGS20
TCEA1
LYPLA1
MRPL15
DRE (A)
SOX17
RP1
CTCF
6
0
H3K27ac
0.3
0.0
HUES64
SOX17Δ5'CTCF#8.2
iPSC
c
SOX17Δ5'CTCF#8.2 - WT
iPSC
Contact difference
10
0
-10
log2FC: -3.38
p-value: 1.9e-35
log2FC: 0.41
p-value: 0.04
CTCF
H3K27ac
HUES64
DRE (A)
SOX17
dEN Differentiation
SOX17Δ5'CTCF#8.2 - WT
dEN
log2FC: -3.33
p-value: 2.1e-41
log2FC: -1.51
p-value: 8.3e-48
3
HUES64 - dEN
d
WT D0 iPSC Rep1
WT D5 dEN Rep3
WT D5 dEN Rep4
WT D5 dEN Rep1
WT D5 dEN Rep2
WT D0 iPSC Rep2
WT D0 iPSC Rep3
WT D0 iPSC Rep4
SOX17Δ5'CTCF#8.2 D0 iPSC Rep4
SOX17Δ5'CTCF#8.2 D5 dEN Rep3
SOX17Δ5'CTCF#8.2 D5 dEN Rep4
SOX17Δ5'CTCF#8.2 D0 iPSC Rep1
SOX17Δ5'CTCF#8.2 D0 iPSC Rep2
SOX17Δ5'CTCF#8.2 D0 iPSC Rep3
SOX17Δ5'CTCF#8.2 D5 dEN Rep1
SOX17Δ5'CTCF#8.2 D5 dEN Rep2
WT D0 iPSCRep4
WT D0 iPSC Rep3
WT D0 iPSC Rep2
WT D5 dEN Rep2
WT D5 dEN Rep1
WT D5 dEN Rep4
WT D5 dEN Rep3
WT D0 iPSC Rep1
0.5
0.6
0.7
0.8
0.9
1.0

Supplementary Fig. 6: cHi-C data analysis of *SOX17* TIG boundary perturbation.
(a) Hi-C contact matrix and CTCF ChIP-seq of the SOX17 locus in ES and iPS cells. Hi-C matrix is provided in 10-kb resolution. CTCF iPSC is GM23338, and Hi-C iPSC is a mixture of reprogrammed cells from 7 individuals from iPSCORE.
(b) Capture Hi-C maps in iPSC wild type (upper panel) and SOX17$^{\Delta 5'CTCF\#8.2}$ (lower panel) at the *SOX17* locus. The normalized capture Hi-C contact maps are overlaid with HUES64 CTCF and H3K27ac ChIP-seq profiles. The deleted centromeric *SOX17* boundary (Boundary 2) is highlighted in grey and marked by a scissor. Putative *SOX17* distal regulatory element DRE (A) and *SOX17* gene body are shown as black bars.
(c) Capture Hi-C subtraction maps in iPSC (upper panel) and dEN (lower panel) at the *SOX17* loop domain. The relative contact difference between the two samples (SOX17$^{\Delta 5'CTCF\#8.2}$ – wild type) in either iPSC or dEN cells are shown on top of HUES64 or HUES64 derived dEN CTCF and H3K27ac ChIP-seq profiles. Boundary 1+2 contact quantifications and contact quantifications between DRE (A) and *SOX17* promoter are highlighted in red circles. The deleted centromeric *SOX17* boundary (Boundary 2) is highlighted in grey marked by a scissor. *SOX17* distal regulatory element DRE (A) and *SOX17* gene body is highlighted in black bars.
(d) Pair-wised pearson correlation between replicates of capture Hi-C data.

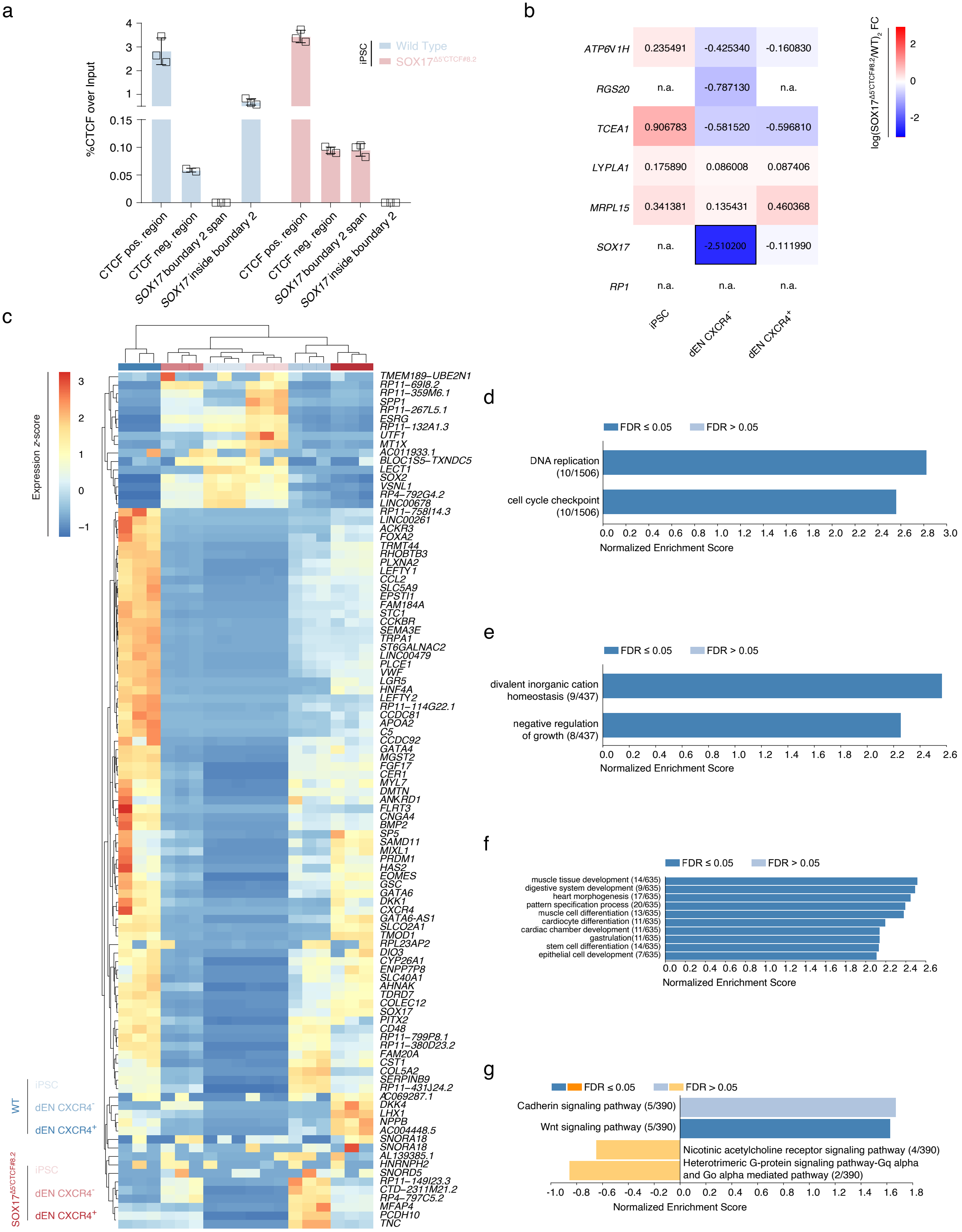
a
%CTCF over Input
4
3
2
1
0.15
0.10
0.05
0
iPSC
Wild Type
SOX17$^{\Delta5'CTCF\#8.2}$
CTCF pos. region
CTCF neg. region
SOX17 boundary 2 span
SOX17 inside boundary 2
CTCF pos. region
CTCF neg. region
SOX17 boundary 2 span
SOX17 inside boundary 2
b
ATP6V1H 0.235491 -0.425340 -0.160830
RGS20 n.a. -0.787130 n.a.
TCEA1 0.906783 -0.581520 -0.596810
LYPLA1 0.175890 0.086008 0.087406
MRPL15 0.341381 0.135431 0.460368
SOX17 n.a. -2.510200 -0.111990
RP1 n.a. n.a. n.a.
iPSC
dEN CXCR4$^-$
dEN CXCR4$^+$
log(SOX17$^{\Delta5'CTCF\#8.2}$/WT)$_2$ FC
2
0
-2
c
Expression z-score
3
2
1
0
−1
TMEM189−UBE2N1
RP11−69I8.2
RP11−359M6.1
SPP1
RP11−267L5.1
ESRG
RP11−132A1.3
UTF1
MT1X
AC011933.1
BLOC1S5−TXNDC5
LECT1
SOX2
VSNL1
RP4−792G4.2
LINC00678
RP11−758I14.3
LINC00261
ACKR3
FOXA2
TRMT44
RHOBTB3
PLXNA2
LEFTY1
CCL2
SLC5A9
EPSTI1
FAM184A
STC1
CCKBR
SEMA3E
TRPA1
ST6GALNAC2
LINC00479
PLCE1
VWF
LGR5
HNF4A
LEFTY2
RP11−114G22.1
CCDC81
APOA2
C5
CCDC92
GATA4
MGST2
FGF17
CER1
MYL7
DMTN
ANKRD1
FLRT3
CNGA4
BMP2
SP5
SAMD11
MIXL1
PRDM1
HAS2
EOMES
GSC
GATA6
DKK1
CXCR4
GATA6-AS1
SLCO2A1
TMOD1
RPL23AP2
DIO3
CYP26A1
ENPP7P8
SLC40A1
AHNAK
TDRD7
COLEC12
SOX17
PITX2
CD48
RP11−799P8.1
RP11−380D23.2
FAM20A
CST1
COL5A2
SERPINB9
RP11−431J24.2
AC069287.1
DKK4
LHX1
NPPB
AC004448.5
SNORA18
SNORA18
AL139385.1
HNRNPH2
SNORD5
RP11−149I23.3
CTD−2311M21.2
RP4−797C5.2
MFAP4
PCDH10
TNC
WT
iPSC
dEN CXCR4$^-$
dEN CXCR4$^+$
SOX17$^{\Delta5'CTCF\#8.2}$
iPSC
dEN CXCR4$^-$
dEN CXCR4$^+$
d
FDR ≤ 0.05
FDR > 0.05
DNA replication (10/1506)
cell cycle checkpoint (10/1506)
0.0 0.2 0.4 0.6 0.8 1.0 1.2 1.4 1.6 1.8 2.0 2.2 2.4 2.6 2.8 3.0
Normalized Enrichment Score
e
FDR ≤ 0.05
FDR > 0.05
divalent inorganic cation homeostasis (9/437)
negative regulation of growth (8/437)
0.0 0.2 0.4 0.6 0.8 1.0 1.2 1.4 1.6 1.8 2.0 2.2 2.4 2.6
Normalized Enrichment Score
f
FDR ≤ 0.05
FDR > 0.05
muscle tissue development (14/635)
digestive system development (9/635)
heart morphogenesis (17/635)
pattern specification process (20/635)
muscle cell differentiation (13/635)
cardiocyte differentiation (11/635)
cardiac chamber development (11/635)
gastrulation(11/635)
stem cell differentiation (14/635)
epithelial cell development (7/635)
0.0 0.2 0.4 0.6 0.8 1.0 1.2 1.4 1.6 1.8 2.0 2.2 2.4 2.6
Normalized Enrichment Score
g
FDR ≤ 0.05
FDR > 0.05
Cadherin signaling pathway (5/390)
Wnt signaling pathway (5/390)
Nicotinic acetylcholine receptor signaling pathway (4/390)
Heterotrimeric G-protein signaling pathway-Gq alpha and Go alpha mediated pathway (2/390)
-1.0 -0.8 -0.6 -0.4 -0.2 0.0 0.2 0.4 0.6 0.8 1.0 1.2 1.4 1.6 1.8
Normalized Enrichment Score

Supplementary Fig. 7: CTCF ChIP-qRT-PCR, GSEA and gene-expression analysis of *SOX17* TIG boundary perturbation.

(a) CTCF ChIP-qRT-PCR of *SOX17* boundary 2 and control regions in undifferentiated SOX17$^{\Delta 5\text{'}CTCF\#8.2}$ or wild type iPSC. CTCF-enrichment is expressed as percentage of CTCF over input, at the respective site of interest (n=3 biological replicates). Data are presented as mean values +/- SD.

(b) TPM expression heatmap of genes associated with the *SOX17* locus. Expression values in iPSC, and CXCR4$^{+/-}$ dEN populations are depicted as log(SOX17$^{\Delta 5\text{'}CTCF\#8.2}$/ WT)$_2$FC.

(c) TPM Z-score raw normalized clustering of the PCA-derived most variable genes (100) across different sub-populations.

(d-f) GO-terms of GSEA for biological processes from different sub-populations. FDRs are depicted by color.

(g) P-terms of GSEA for pathways (PANTHER) from different sub-populations. FDRs are depicted by color.

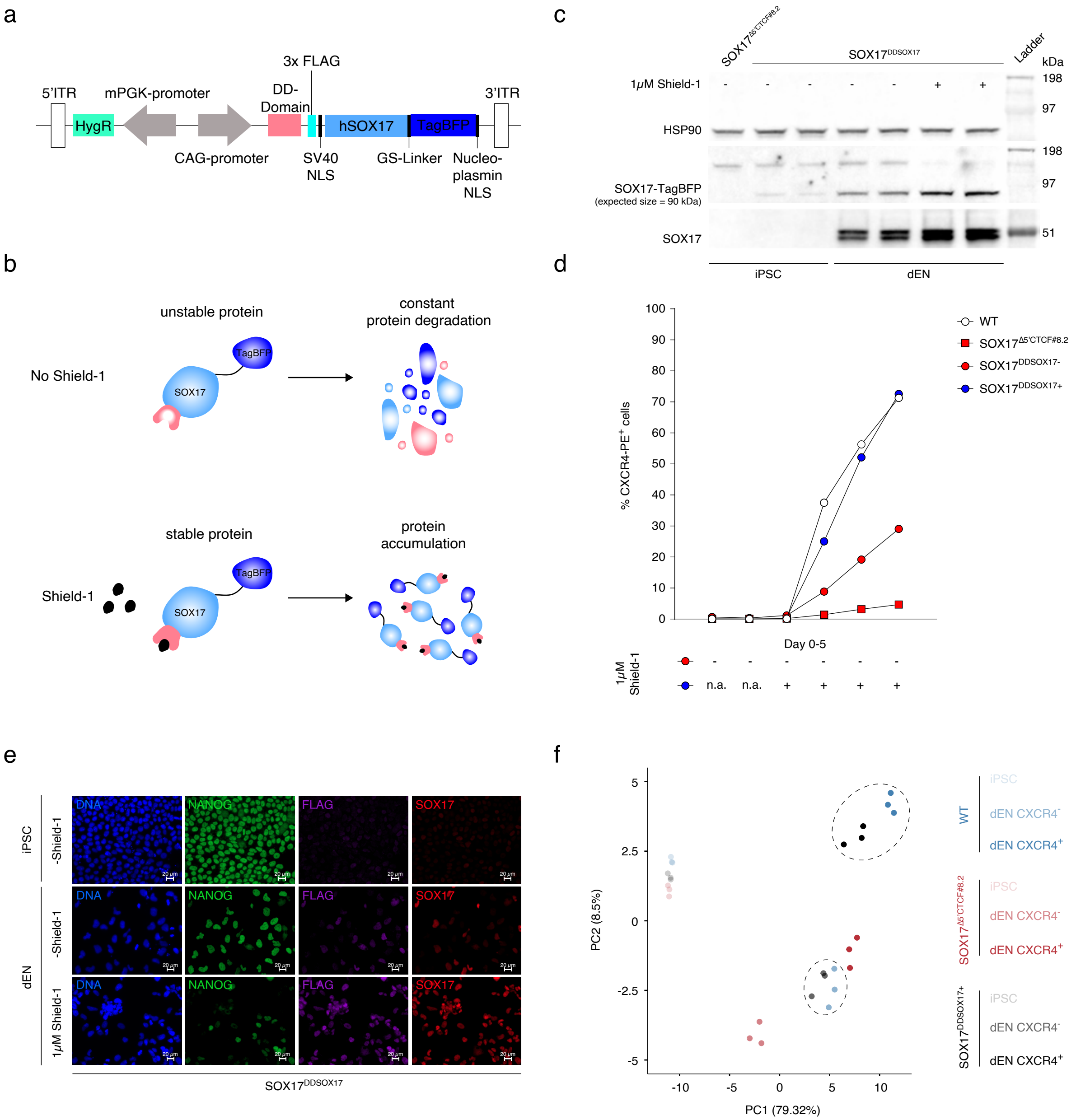

a
3x FLAG
5'ITR
mPGK-promoter
DD-Domain
3'ITR
HygR
hSOX17
TagBFP
CAG-promoter
SV40 NLS
GS-Linker
Nucleo-plasmin NLS
b
unstable protein
constant protein degradation
No Shield-1
TagBFP
SOX17
stable protein
protein accumulation
Shield-1
c
SOX17Δ5'CTCF#8.2
SOX17DDSOX17
Ladder
kDa
1μM Shield-1
- - - - - + +
198
97
HSP90
198
97
SOX17-TagBFP
(expected size = 90 kDa)
SOX17
51
iPSC
dEN
d
% CXCR4-PE+ cells
100
90
80
70
60
50
40
30
20
10
0
Day 0-5
WT
SOX17Δ5'CTCF#8.2
SOX17DDSOX17-
SOX17DDSOX17+
1μM Shield-1
- - - - - -
n.a. n.a. + + + +
e
iPSC
dEN
-Shield-1
-Shield-1
1μM Shield-1
DNA
NANOG
FLAG
SOX17
20 μm
SOX17DDSOX17
f
PC2 (8.5%)
PC1 (79.32%)
5
2.5
0
-2.5
-5
-10
-5
0
5
10
WT
iPSC
dEN CXCR4-
dEN CXCR4+
SOX17Δ5'CTCF#8.2
iPSC
dEN CXCR4-
dEN CXCR4+
SOX17DDSOX17+
iPSC
dEN CXCR4-
dEN CXCR4+

Supplementary Fig. 8: ***SOX17*** **boundary rescue experiments by ectopic SOX17**

(a) Simplified 2D-model of the ITR-flanked piggy-BAC plasmid consisting of constitutively expressed Hygromycin selection marker sequence and inverse directed ectopic SOX17-TagBFP sequence flanked by nuclear localization signal sequence fused to the FKPB12 (L106P mut.) destabilizing domain sequence (DD-Domain).

(b) Schematic of default ectopic SOX17-TagBFP protein degradation and ProteoTuner^{TM} system reversal of protein degradation in presence of Shield-1 compound.

(c) Western Blot data of day 0/5 *in vitro* endoderm differentiated $SOX17^{DDSOX17-}$ (-Shield1) or $SOX17^{DDSOX17+}$ (1µM Shield-1) cell lysates in comparison to $SOX17^{\Delta 5'CTCF\#8.2}$ iPSC for SOX17 and HSP90 protein.

(d) Fluorescence activated cell sorting (FACS) time-course data of wild type, $SOX17^{\Delta 5'CTCF}$ and $SOX17^{DDSOX17-/+}$ iPSC during directed differentiation towards dEN. CXCR4 is depicted as percentage $CXCR4^{+}$ in bulk cell populations. Shield-1 ministered treatment schedule for $SOX17^{DDSOX17+}$ is shown below (n=2 biological replicates). Data are presented as mean values.

(e) Immunofluorescent stainings of NANOG, FLAG, SOX17 and DNA (DAPI) from day 0/5 *in vitro* dEN differentiated $SOX17^{DDSOX17-}$ (-Shield1) or $SOX17^{DDSOX17+}$ (1µM Shield-1) cells.

(f) Principal component analysis of RNA-seq data, depicting sample clusters by the use of the 100 most variable genes. The first two principal components (PCs) are displayed. Dashed lines indicate similarity between $SOX17^{DDSOX17+}$ rescued and wild type dEN cell CXCR4 sub-populations.

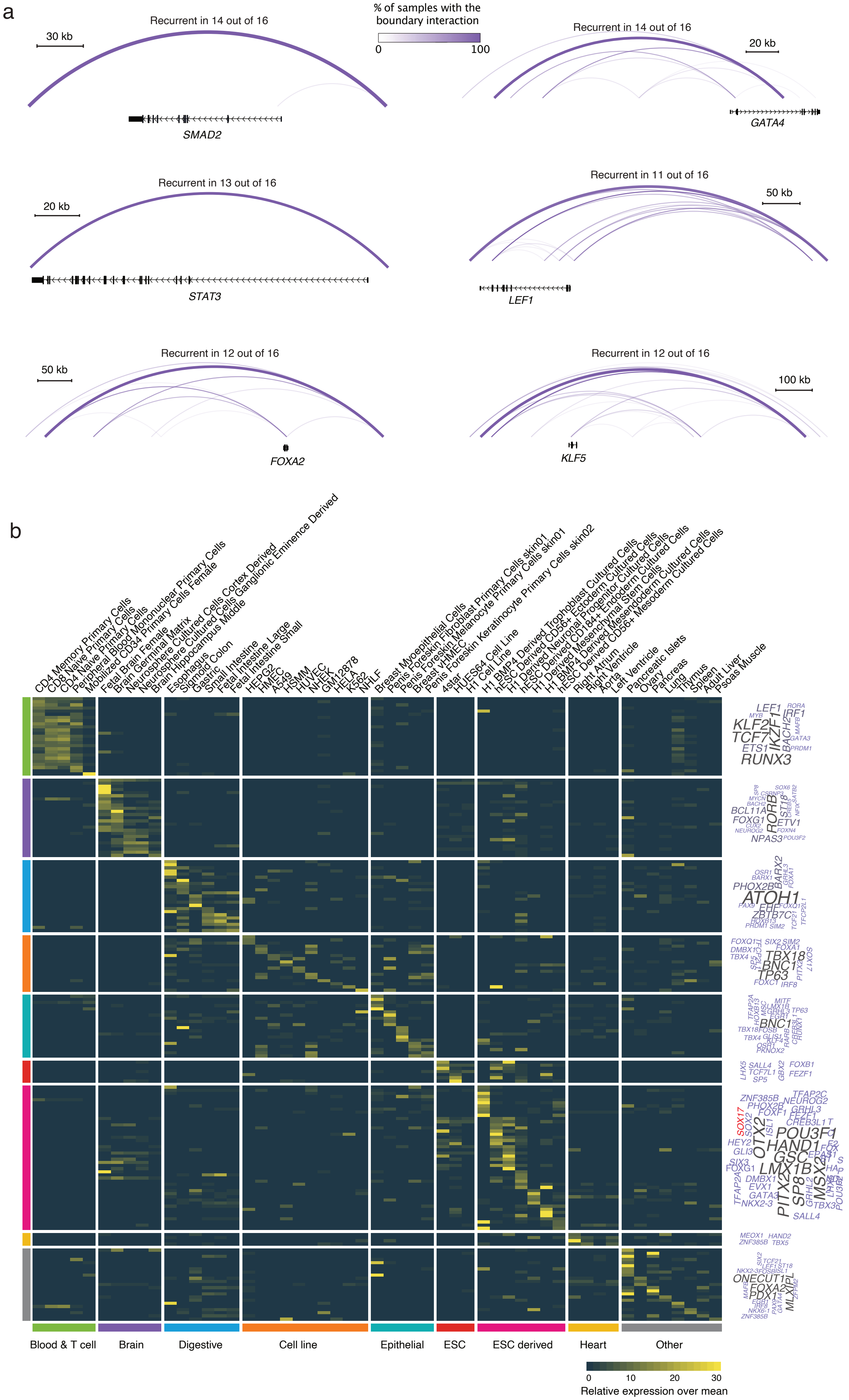
a
% of samples with the boundary interaction
0
100
Recurrent in 14 out of 16
30 kb
SMAD2
Recurrent in 14 out of 16
20 kb
GATA4
Recurrent in 13 out of 16
20 kb
STAT3
Recurrent in 11 out of 16
50 kb
LEF1
Recurrent in 12 out of 16
50 kb
FOXA2
Recurrent in 12 out of 16
100 kb
KLF5
b
CD4 Memory Primary Cells
CD8 Naive Primary Cells
CD4 Naive Primary Cells
Peripheral Blood Mononuclear Primary Cells
Mobilized CD34 Primary Cells Female
Fetal Brain Female
Brain Germinal Matrix
Neurosphere Cultured Cells Cortex Derived
Neurosphere Cultured Cells Ganglionic Eminence Derived
Brain Hippocampus Middle
Esophagus
Sigmoid Colon
Gastric
Small Intestine
Fetal Intestine Large
Fetal Intestine Small
HEPG2
HMEC
A549
HSMM
HUVEC
NHEK
GM12878
HELA
K562
NHLF
Breast Myoepithelial Cells
Penis Foreskin Fibroblast Primary Cells skin01
Penis Foreskin Melanocyte Primary Cells skin01
Breast vHMEC
Penis Foreskin Keratinocyte Primary Cells skin02
4star
HUES64 Cell Line
H1 Cell Line
H1 BMP4 Derived Trophoblast Cultured Cells
hESC Derived CD56+ Ectoderm Cultured Cells
H1 Derived Neuronal Progenitor Cultured Cells
hESC Derived CD184+ Endoderm Cultured Cells
H1 Derived Mesenchymal Stem Cells
H1 BMP4 Derived Mesendoderm Cultured Cells
hESC Derived CD56+ Mesoderm Cultured Cells
Right Atrium
Right Ventricle
Aorta
Left Ventricle
Pancreatic Islets
Ovary
Pancreas
Lung
Thymus
Spleen
Adult Liver
Psoas Muscle
Blood & T cell
Brain
Digestive
Cell line
Epithelial
ESC
ESC derived
Heart
Other
0
10
20
30
Relative expression over mean

Supplementary Fig. 9: Constitutive CTCF loop domains and their functions.

(a) Examples of constitutive domains at the *SMAD2*, *GATA4*, *STAT3*, *LEF1*, *FOXA2* and *KLF5* loci that are conserved across 11-14 out of 16 human cell types. Arcs represent the consensus domains in different cell types with the darker color representing boundary interactions in more cell types. The most recurrent boundary interaction per locus is highlighted using a border line.

(b) Heatmap represents the expression profile of constitutive TIGs that overlap with cell type-specific regulators. Each row represents one gene. The gene names of each group are indicated by a word cloud, in which the frequencies of genes in the different samples of the same group are indicated. Larger font size represents higher frequency thus means the genes are highly expressed in more samples of the same group. Some regulators were recurrent across multiple cell lines of one type, such as *RUNX3* in immune cells, *RORB* in brain tissue, *ATOH1* in digestive tissue and *BNC1* in epithelial cells, while others were specific to one cell type, such as *SOX17* in endoderm and *MYB* in $CD34^+$ cells.

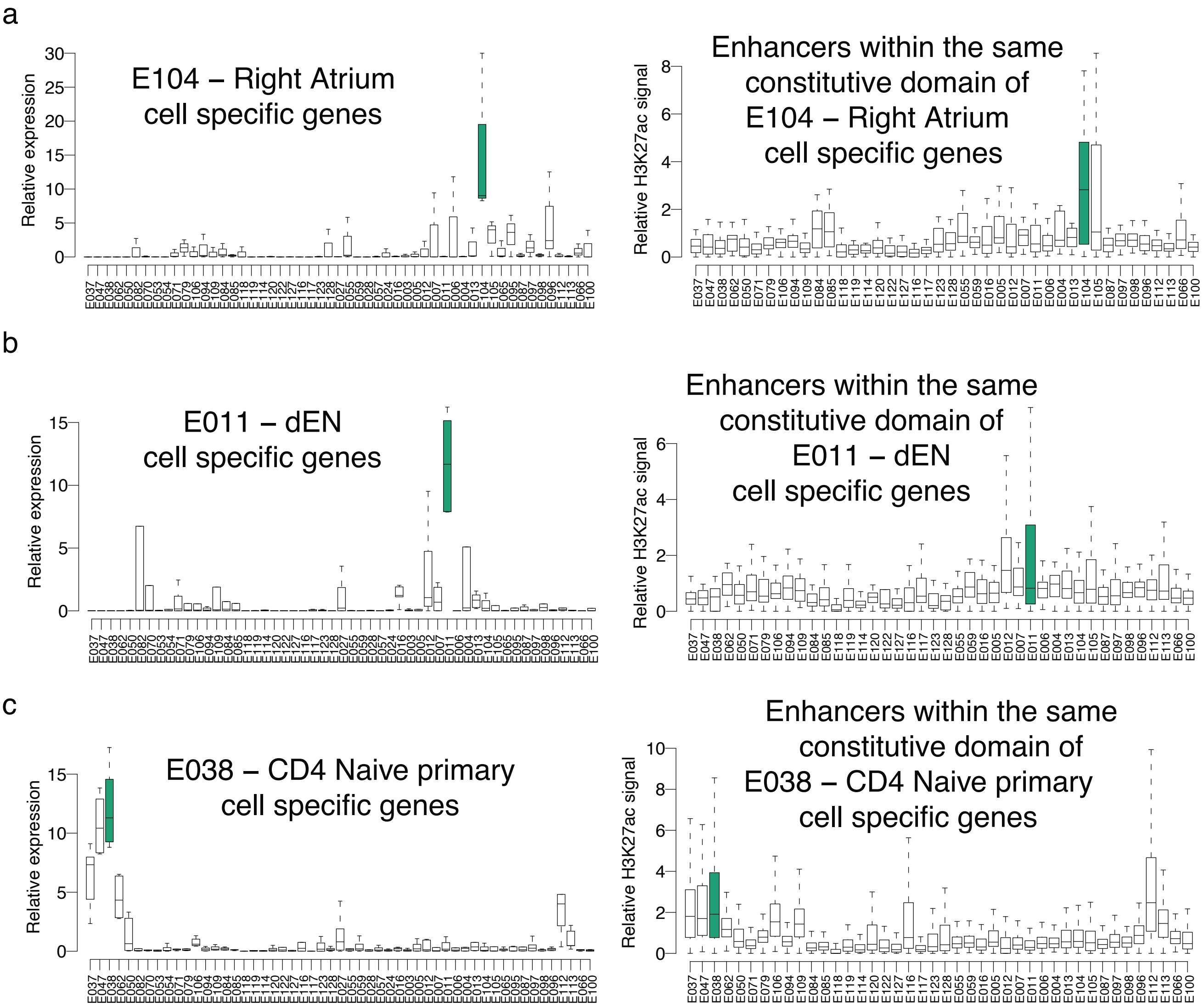
a
E104 – Right Atrium
cell specific genes
Relative expression
Enhancers within the same
constitutive domain of
E104 – Right Atrium
cell specific genes
Relative H3K27ac signal
b
E011 – dEN
cell specific genes
Relative expression
Enhancers within the same
constitutive domain of
E011 – dEN
cell specific genes
Relative H3K27ac signal
c
E038 – CD4 Naive primary
cell specific genes
Relative expression
Enhancers within the same
constitutive domain of
E038 – CD4 Naive primary
cell specific genes
Relative H3K27ac signal

Supplementary Fig. 10: Co-activation patterns between cell type-specific regulators and enhancers insulated by constitutive CTCF loop domains.

(a)-(c) left panel, Relative expression of cell type-specific regulators in E104, E011 and E038 across all cell types. Relative expression is the expression over mean expression across all cell types. The box indicates the interquartile range (IQR), the line inside the box shows the median, and whiskers show the locations of either 1.5 × IQR above the third quartile or 1.5 × IQR below the first quartile, n = 3 genes for E104 (a), n = 6 genes for E011 (b), n = 6 genes for E038 (c).

(a)-(c) right panel, Relative H3K27ac abundance of enhancers located within the same constitutive domain of the cell type-specific regulators in E104, E011 and E038 across all cell types. Relative H3K27ac abundance is the H3K27ac signal over mean H3K27ac signal across all cell types that represents the enhancer activity. The box indicates the interquartile range (IQR), the line inside the box shows the median, and whiskers show the locations of either 1.5 × IQR above the third quartile or 1.5 × IQR below the first quartile, n = 27 enhancers for E104 (a), n = 38 enhancers for E011 (b), n = 166 enhancers for E038 (c).

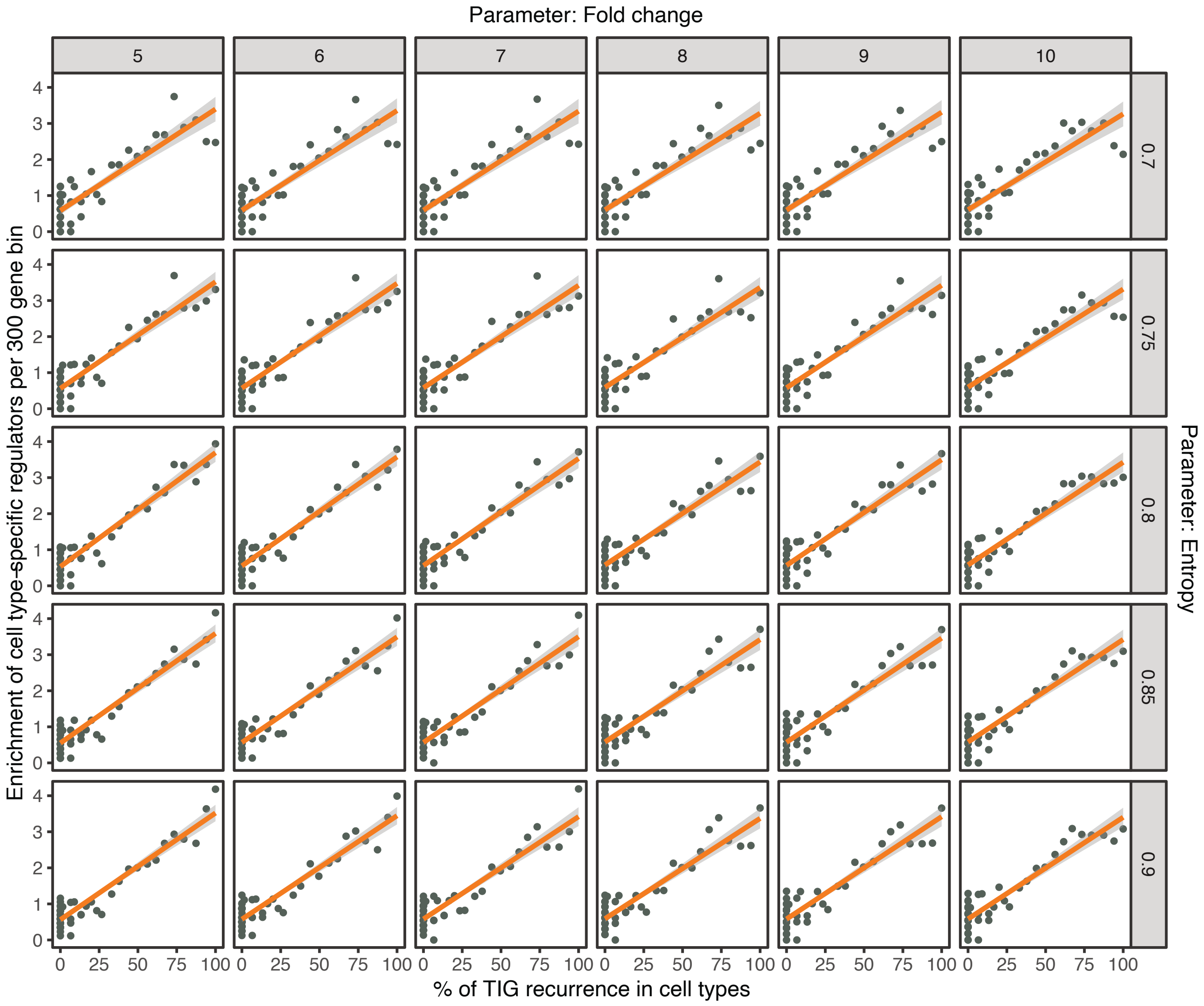
Parameter: Fold change
5
6
7
8
9
10
0.7
0.75
0.8
0.85
0.9
Parameter: Entropy
Enrichment of cell type-specific regulators per 300 gene bin
% of TIG recurrence in cell types
0 1 2 3 4
0 25 50 75 100

Supplementary Fig. 11: **Fold enrichment of cell type-specific regulators against the recurrence of TIGs in multiple cell types.** Each point represents 300 genes. Analysis in Fig. 7a is repeated for different threshold settings for identifying cell type-specific regulators. The results are consistent by changing the entropy setting from 0.7-0.9, and by changing the fold change settings from 5-10. Here fold change represents the case in which a gene has at least x-fold higher expression than the average expression of the gene in all cell types.

Supplementary Table 1. sgRNA sequences for boundary knock-out constructs.

**Oligonucleotide Name**	**Purpose**	**5'-3' Sequence**
SapI_sgRNA_casette_gBlock_+KpnI-sites	Extension cloning of PX458	tgcagacaaatggctctagaggtacggtaccAATATGCATTTTCCCATGATTCCTTCATATTTGCATATACGATACAAGGCTGTTAGAGAGATAATTGGAATTAATTTGACTGTAAACACAAAGATATTAGTACAAAATACGTGACGTAGAAAGTAATAATTTCTTGGGTAGTTTGCAGTTTTAAAATTATGTTTTAAAATGGACTATCATATGCTTACCGTAACTTGAAAGTATTTCGATTTCTTGGCTTTATATATCTTGTGGAAAGGACGAAACACCGGAAGAGCGAGCTCTTCTGTTTTAGAGCTAGAAATAGCAAGTTAAAATAAGGCTAGTCCGTTATCAACTTGAAAAAGTGGCACCGAGTCGGTGCTTTTTTGTTTTCCTGCAGGAGATTTagcgcgtgcgccaattctgcagacaaatggctctagaggtacccgttacataacttacggtaaatggA
AL_SOX17_CTCF_left_gRNA_1	CRISPR/Cas9 Targeting	TGTGGAAAGGACGAAACACCACATCCAGTCTGCCAACATAGTTTTAGAGCTAGAAATAGC
AL_SOX17_CTCF_left_gRNA_2	CRISPR/Cas9 targeting	TGTGGAAAGGACGAAACACCGGGCTGCACCAAATCGCCACGTTTTAGAGCTAGAAATAGC
NANOG_CTCF_right_gRNA_1	CRISPR/Cas9 targeting	TGTGGAAAGGACGAAACACCCATCCTTTGATACACCCCCTGTTTTAGAGCTAGAAATAGC
NANOG_CTCF_right_gRNA_2	CRISPR/Cas9 targeting	TGTGGAAAGGACGAAACACCTGTTAGTCTTTGTTCAACCGGTTTTAGAGCTAGAAATAGC

Supplementary Table 2. Oligonucleotides used for genotype verification.

Oligonucleotide Name	Purpose	hg19 Coordinates	5'-3' Sequence
NANOG_spanbound_fwd	Genotyping	chr12:7958132-7958151	GCCAGGGAGATAGGTGTGAA
NANOG_spanbound_rev	Genotyping	chr12:7960762-7960781	GACTAGCTCACGTCATCCCC
NANOG_int_fwd	Genotyping	chr12:7959257-7959278	GGTTGTGATCCCATTCATGTCA
NANOG_int_rev	Genotyping	chr12:7959726-7959745	TCCCTTGTCCCTGAACATGT
SOX17_spanbound_fwd	Genotyping	chr8:55077369-55077388	GCTCTGCACGTGGTAAAAGA
SOX17_spanbound_rev	Genotyping	chr8:55083240-55083259	TGAAGAGGACCATGAGCACA
SOX17_int_fwd	Genotyping	chr8:55079982-55080001	ACACGCTAAGCCACAATGAG
SOX17_int_rev	Genotyping	chr8:55080374-55080393	TTCTTCACAACCTTGCCAGC
pJet1.2_fwd	Genotyping	n.a.	CGACTCACTATAGGGAGAGCGGC
pJet1.2_rev	Genotyping	n.a.	AAGAACATCGATTTTCCATGGCAG

Supplementary Table 3. Antibodies used for FACS.

Antibody name	Application / Dilution used	Clone / Company	Catalogue number
Mouse anti-Human CRCX4 (CD184) PE	FACS / 1:200	12G5 / Biolegend	306506
Mouse IgG2aκ PE Isotype control	FACS / 1:200	MOPC-173 / Biolegend	400212
Mouse anti-Human Ep-CAM (CD326) PE	FACS / 1:200	9C4 / Biolegend	324206
Mouse IgG2bκ PE Isotype control	FACS / 1:200	MPC-11 / Biolegend	400314
Mouse IgG1κ AlexaFluor488 Isotype control	FACS / 1:50	MOPC-21 / BD	557702
Mouse AlexaFluor488 SOX17	FACS / 1:50	P7-969 / BD	562205
Mouse AlexaFluor488 NANOG	FACS / 1:50	N31-355 / BD	560791

Supplementary Table 4. Antibodies used for Immunofluorescence staining and Western Blot.

Antibody name	Application / Dilution used	Clone / Company	Catalogue number
Rabbit anti-Human NANOG (unconjugated)	1st IF / 1:1000	EPR2027(2) / Abcam	ab109250
Goat anti-Human SOX17 (unconjugated)	1st IF & WB / 1:1000	Polyclonal / R&D Systems	AF1924
Mouse anti-Human OCT4 (unconjugated)	1st IF / 1:2000	Polyclonal / Abcam	ab19857
Mouse anti-FLAG® M2 (unconjugated)	1st IF / 1:1000	M2 / Sigma	F1804-50UG
Donkey anti-Mouse IgG AlexaFluor488	2nd IF / 1:700	Polyclonal / Thermo Fisher Scientific	A21202
Donkey anti-Goat IgG AlexaFluor594	2nd IF / 1:700	Polyclonal / Thermo Fisher Scientific	A11058
Donkey anti-Rabbit IgG AlexaFluor647	2nd IF / 1:700	Polyclonal / Thermo Fisher Scientific	A31573

Supplementary Table 5. Antibodies only used for Western Blot.

**Antibody name**	**Application**	**Clone / Company**	**Catalogue number**
Mouse anti-Human HSP90 (unconjugated)	1st WB / 1:4000	68/Hsp90 / BD	610419
Donkey anti-Mouse IgG HRP	1st WB / 1:5000	Polyclonal / Jackson ImmunoResearch	715-035-150
Donkey anti-Goat IgG HRP	1st WB / 1:5000	Polyclonal / Jackson ImmunoResearch	705-035-147

Supplementary Table 6. Oligonucleotide sequences used for CTCF ChIP qRT-PCR.

Oligonucleotide Name	Purpose/ Region	hg19 Coordinates	5'-3' Sequence
CTCF_ChIPqPCR_spanbound_fwd	ChIP qRT-PCR /KO span-region	chr8:55077729-55077748	GTGCCCTCCCCAAAACATTT
CTCF_ChIPqPCR_spanbound_rev	ChIP qRT-PCR /KO span-region	chr8:55083139-55083158	GCCTGCTCTCAAAACCTTCA
CTCF_ChIPqPCR_int_fwd	ChIP qRT-PCR /CTCF-motif 2	chr8:55082039-55082059	TGCAGTACCACATCTTGAACA
CTCF_ChIPqPCR_int_rev	ChIP qRT-PCR /CTCF-motif 2	chr8:55082114-55082133	GCAAAACAACTTACAGCGGC
CTCF_ChIPqPCR_neg_fwd	ChIP qRT-PCR /neg. ctrl. region	chr8:55262554-55262573	TTGAGTCCCAGAGGTTGAGG
CTCF_ChIPqPCR_neg_rev	ChIP qRT-PCR / neg. ctrl. region	chr8:55262609-55262628	GTCTCACTTTGTCCCCTGGG
CTCF_ChIPqPCR_pos_fwd	ChIP qRT-PCR / pos. ctrl. region	chr8:55464418-55464437	GCCTTCAAAGCGGGTCATTT
CTCF_ChIPqPCR_pos_rev	ChIP qRT-PCR / pos. ctrl. region	chr8:55464477-55464496	AACCCTCACAAACCCAGACA
